# Evaluation of prostate cancer pathology reports generated by ChatGPT to enhance patient comprehension

**DOI:** 10.1038/s41598-025-17639-1

**Published:** 2025-10-02

**Authors:** Narmina Khanmammadova, Rafael Gevorkyan, Andrew S. Afyouni, Andrei D. Cumpanas, Daniel Jiang, Kristene Myklak, Tuan Thanh Nguyen, Sohrab N. Ali, Jacob Basilius, Antônio Rebello Horta Görgen, Jaime Altamirano-Villarroel, Mohammed Shahait, Michael A. Daneshvar, Thomas E. Ahlering, David I. Lee

**Affiliations:** 1 Department of Urology, University of California, Irvine, CA USA; 2 Keck School of Medicine, University of Southern California, Los Angeles, CA USA; 3 Department of Urology, University of Medicine and Pharmacy at Ho Chi Minh City, Ho Chi Minh, Vietnam; 4 Department of Urology, Hospital de Clínicas de Porto Alegre, Porto Alegre, Brazil; 5 Department of Urology, Fundación Arturo López Pérez, Santiago, Chile; 6 Division of Urology and Urologic Oncology, Department of Surgery, City of Hope, Duarte, CA USA; 7 Urology, Golden State Urology, Fremont, CA USA; 8 Urology, Intermountain Health, Heber City, UT USA

**Keywords:** ChatGPT, Patient-centered pathology reports, Prostate cancer, AI-generated reports, Prostate needle biopsy, Large language models, Prostate cancer, Prostate cancer

## Abstract

The technical language of medical reports can be difficult for laypeople to understand. Artificial intelligence (AI) tools such as OpenAI’s ChatGPT present an opportunity to bridge this gap. This quality improvement initiative explored ChatGPT’s potential to translate traditional prostate cancer pathology reports into patient-friendly versions. Using institutional templates, we created 25 prostate biopsy and 25 radical prostatectomy reports and converted them into patient-centered versions using ChatGPT-3.5. Nine urologists evaluated these reports. Additionally, 41 patients participating in the initiative were asked to provide feedback on their understanding of the biopsy reports. Feedback was collected using structured questionnaires and readability scores. The AI-generated biopsy and surgical pathology reports had higher mean Flesch Reading Ease Scores (49 ± 5; 50 ± 3) compared to the original reports (31 ± 15, *p* < 0.001; 41 ± 17, *p* = 0.02, respectively). Surgeons expressed positive opinions about AI-generated biopsy and surgical pathology reports, including patient-friendly language (77% and 95%, respectively). Patients also agreed that the AI-generated biopsy reports were well-structured and easy to follow (90%) and helpful in understanding their diagnosis (88%). 76% of patients preferred AI-generated reports as part of their care. These findings suggest that AI-developed reports may improve readability and support patient understanding.

**Supplementary Information:**

The online version contains supplementary material available at 10.1038/s41598-025-17639-1.

## Introduction

The term artificial intelligence (AI) typically denotes computational technologies that imitate or replicate intellectual functions characteristic of human cognitive processes, including logical thinking, learning, and problem-solving. Thus, AI has extensive and growing computational capability, facilitating task performance and quickly revolutionizing different fields of science, including medicine^1^. Recently, AI and Chat Generative Pretrained Transformer (ChatGPT) have significantly contributed to the advancement and capability of Large Language Models (LLMs) in healthcare practices. Beyond improving the quality of care, the use of AI in clinical practice holds promise for enhancing patient comprehension as health literacy remains a major barrier in healthcare communication. Reports show that at least one-third of adults in the United States (US) have low health literacy^2–4^. This is particularly concerning given that, as Mossanen et al. reported, pathology reports for various urologic cancer procedures have high readability indices, with radical prostatectomy and prostate biopsy reports being among the most complex, making them difficult for laypersons to interpret^5^. Considering that prostate cancer is the most common solid cancer among men in the US^6^, it is important to explore whether freely accessible AI platforms are reliable and can contribute to improving patient comprehension of the cancer diagnosis and disease characteristics.

ChatGPT is a chatbot developed by OpenAI (San Francisco, California, USA) and has a publicly available version – ChatGPT 3.5. To date, ChatGPT has been evaluated in different facets of urologic practice, such as helping with administrative work (i.e., discharge notes, documentation), research, clinical performance to simplify the process of diagnosis and treatment decision, and engagement of patients in shared decision-making^6–10^. While there is uncertainty about the accuracy of the information delivered, patients are already relying on such tools to understand the details of radiologic examinations and procedural outcomes^11^.

This initiative aimed to evaluate surgeons’ insights regarding the accuracy and comprehensiveness of the information provided by ChatGPT. We also explored the potential usability of AI-generated prostate needle biopsy (PBx) and radical prostatectomy (RP) surgical pathology reports. Additionally, as part of a quality improvement initiative focusing on enhancing patient-physician communication, we examined patients’ understanding of the AI-generated reports and their willingness to receive this kind of assistance during their care.

## Materials and methods

### Project design

This project was conducted as a quality improvement initiative at the University of California, Irvine (UCI), aimed at enhancing patient care delivery through improved patient–provider communication. In accordance with the policies of the UCI Institutional Review Board (IRB), quality improvement activities of this nature do not constitute human subjects research and therefore do not require IRB review. Investigator-developed questionnaires were used to assess the utility of ChatGPT 3.5 in generating patient-centered reports from traditional pathology reports. This initiative was conducted in accordance with institutional regulations, and informed consent was obtained from all participants.

Institutional templates from twenty-five original PBx and 25 RP pathology reports were utilized to develop sample reports that could be input into ChatGPT. Of note, no reports involving patient data were used for this initiative. The reports were sampled equally across Gleason Grade groups (GGG) to encompass varying degrees of prostate cancer severity. Surveys for surgeons and patients were developed based on similar criteria addressing specific aspects of the reports, such as accuracy, readability, and clinical utility. Both urologists and patients provided feedback in the form of structured questionnaires utilizing a Likert Scale (1 – strongly disagree, 2 – somewhat disagree, 3 – neutral, 4 – somewhat agree, 5 – strongly agree) for responses.

The primary aim of this initiative was to gather surgeons’ and patients’ insights regarding the ChatGPT-generated reports. We also reviewed the readability of these new reports relative to the reports that were input into ChatGPT.

### Report generation

The institutional pathology report templates were used, and sample reports were generated representing all GGG groups. These reports are referred to as “original” reports throughout the manuscript. Using the ChatGPT-3.5 model, the original prostate needle biopsy and surgical pathology reports were converted into patient-centered reports. Specific prompts were provided to ChatGPT to ensure the generation of reports utilizing patient-friendly language. The prompts for each respective report are shown in Supplementary Materials 1 and 2.

### Surgeon surveys - report evaluation

Nine urologists from 5 different countries (US, Vietnam, United Arab Emirates, Brazil, and Chile) reviewed new AI-generated PBx and RP pathology reports. Urologists who participated in this initiative had various levels of experience (e.g., early career, mid-career, and surgeons with > 25 years of experience), and each urologist had experience in diagnosing and treating prostate cancer patients. Custom-designed 5-point Likert scale questionnaires consisting of 7 statements for PBx and 8 statements for RP reports, ranging from “1- Strongly disagree” to “5 - Strongly agree” were employed to gather feedback on various aspects of the reports. The following criteria were used to develop the surveys: structure, readability, accuracy, explanation of the disease extent, Gleason grade interpretation, comparative effectiveness, and clinical utility. (Table 1) For RP reports, there was an extra criterion evaluating surgical margin explanation as well. (Table 1)

**Table 1:** Surgeon evaluation breakdown for ChatGPT-generated biopsy and surgical pathology reports based on questionnaire criteria.

ChatGPT-generated biopsy reports					
Questions:	Strongly disagree	Somewhat disagree	Neutral	Somewhat agree	Strongly agree
Structure: 1. The ChatGPT report is well-structured and easy to follow.	0 (0%)	3 (1%)	67 (30%)	115 (51%)	40 (18%)
Readability: 2. The report clearly states the diagnosis in terms that a patient could easily understand.	1 (1%)	7 (3%)	43 (19%)	77 (34%)	97 (43%)
Accuracy: 3. The report correctly describes the biopsy results based on the given data.	0 (0%)	7 (3%)	12 (5%)	93 (42%)	113 (50%)
Disease extent explanation: 4. The report explains the extent of the disease accurately.	0 (0%)	13 (6%)	28 (12%)	69 (31%)	115 (51%)
Gleason grade interpretation: 5. The report interprets Gleason grade accurately.	2 (1%)	4 (2%)	12 (5%)	89 (40%)	118 (52%)
Comparative effectiveness: 6. Compared to the original report, the ChatGPT report will improve patients’ understanding of their disease.	0 (0%)	6 (3%)	53 (24%)	120 (53%)	46 (20%)
Clinical utility: 7. You are willing to use patient-centered generative AI reports in your practice.	0 (0%)	2 (1%)	8 (4%)	75 (33%)	140 (62%)

ChatGPT-generated surgical pathology reports:					
Questions:	Strongly disagree	Somewhat disagree	Neutral	Somewhat agree	Strongly agree
Structure: 1. The ChatGPT report is well-structured and easy to follow.	0 (0%)	0 (0%)	9 (4%)	75 (33%)	141 (63%)
Readability: 2. The report correctly explains complex medical terms and procedures in patient-friendly language.	0 (0%)	0 (0%)	11 (5%)	58 (26%)	156 (69%)
Accuracy: 3. The report correctly describes the pathology results based on the given data.	0 (0%)	1 (1%)	43 (19%)	23 (10%)	158 (70%)
Disease extent explanation: 4. The report explains the extent of the disease accurately (pathological stage, extraprostatic extension, seminal vesicle invasion, and lymph node involvement).	0 (0%)	1 (1%)	17 (7%)	96 (43%)	111 (49%)
Gleason grade interpretation: 5. The report interprets Gleason grade accurately.	0 (0%)	0 (0%)	27 (12%)	60 (27%)	138 (61%)
Surgical margin explanation: 6. The report explains surgical margins accurately.	0 (0%)	15 (7%)	34 (15%)	41 (18%)	135 (60%)
Comparative effectiveness: 7. Compared to the original report, the ChatGPT report will improve patients’ understanding of their disease.	0 (0%)	1 (1%)	4 (2%)	97 (43%)	123 (54%)
Clinical utility: 8. You are willing to use patient-centered generative AI reports in your practice.	0 (0%)	0 (0%)	2 (1%)	59 (26%)	164 (73%)

### Patient surveys - report evaluation

Participation criteria included willingness to participate, having a diagnosis of prostate cancer, and the ability to read and speak English. Being a native English speaker was not a requirement. Fifty consecutive patients (September 2023 to October 2023) were approached. Forty-one (82%) patients decided to participate and complete the questionnaires. Patients were at different stages of their treatment for prostate cancer, and all received randomly distributed one ChatGPT-generated PBx report in an outpatient setting. It was clearly explained that the reports provided for this initiative do not represent their personal clinical data. A sample of the original pathology report was also provided to participants alongside the AI-generated reports for comparison.

Patient surveys consisted of 6 statements. Tailored 5-point Likert scale surveys, spanning from “1 - Strongly disagree” to “5 - Strongly agree” were utilized to gather feedback on the quality of the reports. The following criteria and questions were used to explore patients’ perspectives: structure, readability, disease extent explanation, Gleason grade explanation, comprehension, and future usability. (Table 2)

**Table 2:** Patients’ perspective breakdown for ChatGPT-generated biopsy reports.

Questions:	Strongly disagree	Somewhat disagree	Neutral	Somewhat agree	Strongly agree
Structure: 1. This report is well-structured and easy to follow.	0 (0%)	2 (5%)	2 (5%)	6 (14%)	31 (76%)
Readability: 2. It clearly states the diagnosis, and I can understand the diagnosis easily.	0 (0%)	1 (2%)	4 (10%)	7 (17%)	29 (71%)
Disease extent explanation: 3. I can understand the extent of the disease easily.	0 (0%)	1 (2%)	5 (12%)	8 (20%)	27 (66%)
Gleason grade explanation: 4. The Gleason grade of cancer is clear to me.	0 (0%)	2 (5%)	4 (10%)	11 (27%)	24 (58%)
Comprehension: 5. This report will improve my understanding and other patients’ understanding of the disease.	0 (0%)	0 (0%)	7 (17%)	8 (20%)	26 (63%)
Future usability: 6. I would like to receive patient-centered generative AI reports during my care.	3 (7%)	0 (0%)	7 (17%)	6 (14%)	25 (62%)

### Readability assessment

Validated readability metrics were employed to assess the comprehensibility of the report. In our case, the readability of the AI-generated reports was evaluated by employing two validated readability formulas: the Flesch-Kincaid grade level and the Flesch Reading Ease score^12^. These formulas calculate readability based on the average sentence length (measured as the number of words utilized) and average word length (measured as the number of syllables within each word). However, the scales of the two metrics are different.

The Flesch Reading Ease score quantifies readability on a scale of 0 to 100, with higher scores indicating easier readability. Scores of 60–70 are considered standard and easily understood by 13- to 15-year-old students. For scores below 30, the sentence structure is more complex, similar to that encountered when reading college-level texts.

Flesch Reading Ease score = 206.835–0.846 x number of syllables per 100 words – 1.015 x average number of words per sentence^13^.

The Flesch-Kincaid Grade Level scale equates readability with the US grade levels. For instance, a score of “6” would mean that the text is suitable for a 6th grader. Previous studies have suggested that a piece of written medical information made for a patient with a low level of literacy should have a score of ≤ “8” to ensure adequate comprehension of the presented medical information.

Flesch Kincaid Grade Level = 0.39 x average number of words per sentence + 11.8 x number of syllables per word − 15.59^13^.

These metrics allowed for a more direct and objective comparison of the readability of the original and ChatGPT-generated reports.

IBM SPSS Statistics for Windows version 29.0 (IBM Corp, Armonk, NY, USA) was used for statistical analysis. Categorical variables are represented as proportions and percentages. Student’s t-test was utilized for continuous variables; a p-value < 0.05 was considered significant.

## Results

### Readability metrics

ChatGPT-generated biopsy reports (Supplementary Material 1) had a Flesch Reading Ease Score of 49 ± 5 as compared to the original reports’ score of 31.2 ± 14.6 (*p* < 0.001). This was similar for surgical pathology reports (Supplementary Material 2); the Flesch Reading Ease Score was higher in the ChatGPT-generated reports (49.6 ± 3.4) in contrast to the original reports (41.4 ± 16.8; *p* = 0.020).

Meanwhile, the Flesch-Kincaid Grade Level was higher in the ChatGPT-generated reports (10.5 ± 0.7) than in the original reports (8.7 ± 2.2; *p* < 0.001). The Flesch-Kincaid Grade Levels were similar between the two versions of PBx reports (*p* = 0.940).

### Surgeon evaluations

All surgeons completed the evaluation of the 50 AI-generated reports using a structured questionnaire utilizing a Likert scale. When reviewing the structure and clarity of the ChatGPT-generated reports, surgeons agreed (either somewhat or strongly) that the new AI-generated patient-centered reports were well-structured and easy to follow (PBx: 69%; surgical pathology: 96%). Surgeons also agreed (either somewhat or strongly) that patients would likely easily understand the diagnosis from these reports (PBx: 77%; surgical pathology: 95%).

In terms of accuracy, there was 92% agreement (somewhat and strongly) in the PBx report evaluations and 80% in the surgical pathology report evaluations (Table 1) that the ChatGPT reports provided an accurate description of the pathology results based on the provided data.

Regarding the willingness to use AI-generated reports in clinical practice, 95% of new PBx reports and 99% of new surgical pathology reports were viewed as potentially useful tools in clinical settings.

### Patient evaluations

To understand urology patient perspective on ChatGPT-generated PBx reports, 41 patients were approached during clinic visits within 2 months. The mean ± SD age of the cohort was 66.8 ± 9.1 years, and all patients were enrolled at the Urology clinic at the tertiary care academic medical center. The racial composition of the cohort was as follows: 46.3% White (*n* = 19), 29.3% Asian (*n* = 12), 7.3% African American (*n* = 3), and 17.1% self-identified as mixed or other (*n* = 7). There were no patient evaluations with missing responses to the Likert-scale items. (Table 2)

90% of the patients found the AI-generated reports to be well-structured and easy to follow (*n* = 37/41). When asked about the clarity of the report, 88% (*n* = 36/41) of the patients agreed that it was able to convey the diagnosis and make it easy to interpret. 86% of patients felt that the report was informative in explaining the extent of the disease (*n* = 35/41), and 83% (*n* = 34/41) mentioned that it was clear in explaining the GGG. 76% of the patients (*n* = 31/41) expressed that they would be interested in receiving future AI-generated reports that are curated to their own health needs.

## Discussion

As part of a quality improvement project, we used ChatGPT to generate patient-centered pathology reports for PBx and RPs. We aimed to explore the potential role of LLM in simplification of these reports, as the presented information on these reports is complex and challenging to understand for an average person.

Surgeons graded AI-generated reports as patient-friendly (PBx, 77%; surgical pathology, 95%), and well-structured (PBx: 69%; surgical pathology: 96%). Both surgeons (PBx: 95%; surgical pathology: 99%) and a majority of urology patients (76%) showed an interest in utilizing these new reports as adjunctive materials in clinical settings.

The increase in the Flesch Reading Ease score for the ChatGPT-generated PBx reports suggests that the model may help convey complex medical information more understandably. The similar Flesch-Kincaid Grade levels between the original and generated PBx reports suggest that the complexity of the content may have remained largely unchanged, maintaining the necessary depth of information. Surgical pathology reports mirrored these findings, with an increase in the readability scores of the ChatGPT-generated reports. There was a slight increase in the Flesch-Kincaid Grade Level observed. This persistent complexity is likely attributable to the inherently technical nature of PBx and RP reports, which are consistently reported to be difficult to simplify – even after the manual removal of complex medical terms by healthcare providers – as demonstrated by Mossanen et al., with Flesch-Kincaid Grade Levels consistently at or above 10^5^.

While AI holds the potential to enhance current medical practices, a significant concern is its potential to yield misinformation in the healthcare setting, as it has limited access to reliable, updated resources^14,15^. In our quality improvement initiative, we observed that ChatGPT can handle the interpretation of accurately presented complex medical data with a well-written prompt. In 82% of the PBx survey responses, surgeons agreed (strongly and somewhat) that the explanation of the disease extent was accurate. Additionally, in 80% of survey responses, surgeons agreed (strongly and somewhat) to the accuracy of new surgical pathology reports. They also agreed (strongly and somewhat) that the Gleason grade interpretation was precise in 92% of the PBx survey responses and 88% of the surgical pathology survey outcomes. These findings appear slightly lower than those reported in a study by Proctor et al., in which all PBx reports were deemed accurate (completely or mostly correct) by both urologists and pathologists^16^. The modest discrepancy of 20% may be due to contextual differences, including the use of a more advanced version of ChatGPT (version 4.0), variations in prompting, or differences in user surveys. Notably, although all reports were classified as complete, grammatically correct, and accurate, urologists in the Proctor et al. study reported concerns regarding the severe potential for patient harm in 20% of ChatGPT-generated reports, with the level of concern increasing linearly with disease risk^16^. Taken together, these observations reinforce the importance of human oversight, as some inaccuracies or incomplete disease explanations may still require clarification by healthcare providers.

Furthermore, utilizing ChatGPT could enhance disease comprehension among patients, especially those from lower socio-economic backgrounds and with limited health literacy. Previous literature demonstrated that these factors have a significant impact on the cancer stage at diagnosis, treatment selection, and survival outcomes^16–18^. However, the use of plain and personalized language significantly improves comprehension of evidence-based scientific knowledge and increases subsequent patient engagement^19,20^. As part of this initiative, surgeons agreed (strongly and somewhat) that ChatGPT reports might aid in understanding the disease (biopsy: 73%; surgical pathology: 97%), and patients (76%, somewhat and strongly agree) showed interest in utilizing these new reports as supplementary material. Further evaluation may help clarify the effect of AI-generated reports on long-term outcomes, such as choice of treatment.

In modern healthcare, the significance of health literacy and effective patient-centered communication cannot be overstated. However, the challenge lies in the scarcity of accurate, precise, and easily understandable medical information. Various healthcare organizations have suggested different readability benchmarks for patient-oriented materials. For instance, the American Medical Association recommends that patient-dedicated materials be written at or below a 6th-grade reading level^21^. Conversely, the Centers for Disease Control and Prevention recommends materials be written at no higher than an 8th-grade reading level^22^. Interestingly, although ChatGPT 3.5-generated reports showed a notable improvement in readability compared to the originals, they still did not meet the standards set by these organizations. Despite its potential as a complementary tool in clinical care, it remains a significant distance away from replacing clinician guidance. Given these findings, it is paramount to emphasize that if used in clinical settings, ChatGPT-generated patient-centered reports should be reviewed by healthcare professionals prior to patient distribution, as similarly recommended by previous literature^16,23^.

Although this is the first initiative to explore the readability and perceived benefit of ChatGPT in this particular setting, we acknowledge several limitations. The main limitation of this project was the small patient cohort and the evaluation by a limited number of urologists. Additionally, we chose to utilize the publicly available version, ChatGPT 3.5, whereas the most recent version, ChatGPT 4, demonstrates significantly improved precision and comprehensiveness – particularly in tasks such as reasoning, language processing, and responding to complex questions^30^. Further exploration using more advanced versions of ChatGPT may help assess its potential to improve patient understanding and engagement. Of note, we did not observe any hallucinations. However, we acknowledge that to comprehensively evaluate the range of potential behaviors exhibited by LLMs, a greater number of reports encompassing diverse disease characteristics should be assessed. We also recognize the inherent variability in chatbot outputs, even when using identical prompts, which may limit reproducibility and consistency of responses.

Certain ethical rules and codes of conduct for the use of AI in healthcare are currently being developed and should be considered when implementing or utilizing AI bots for medical reports in clinical settings^24–29^. Several principles are widely endorsed, including the four principles of medical ethics – beneficence, nonmaleficence, autonomy, and justice – as well as transparency, accountability, and dependability. Publicly available chatbots fall outside the domain of regulated healthcare AI and are, in most cases, not subject to oversight by regulatory entities. When such tools are used in clinical settings, physicians should adhere to institutional policies and ensure appropriate oversight. Similarly, the Federation of State Medical Boards recommends that physicians directly supervise the use of AI in healthcare and remain accountable for any resulting harm^30^. As such, educating patients about the potential for misleading medical information generated by these platforms is emphasized, and the results of this initiative should be applied with caution, taking these considerations into account.

## Conclusion

The integration of LLMs in healthcare, particularly patient communication, has transformative potential. This initiative focuses on the capabilities of the ChatGPT-3.5 language model and suggests its potential to enhance the readability and comprehensibility of medical reports in the realm of prostate cancer.

Feedback from urologists suggests that the model may be able to generate reports with improved understandability while simultaneously maintaining the accuracy of original clinical reports. The majority of surgeons expressed willingness to incorporate AI-generated reports into their practice, indicating a significant endorsement of its utility. Similarly, approximately three-fourths of patients expressed interest in receiving these revised reports as supplementary materials during their care, further underscoring the model’s potential clinical value. However, there are areas for refinement, particularly in the explanation of surgical margins and GGG interpretations. This feedback serves as a valuable roadmap for the continued development and optimization of the utilization of AI tools in this domain.

## Supplementary Information

Below is the link to the electronic supplementary material.

Supplementary Material 1

## Data Availability

The datasets generated during and/or analyzed during the current project are not publicly available due to institutional data-sharing restrictions, but are available from the corresponding author on reasonable request.

## Abbreviations

AI: Artificial intelligence
ChatGPT: Chat generative pretrained transformer
GGG: Gleason grade group
IRB: Institutional review board
LLM: Large lange models
PBx: Prostate biopsy
RP: Radical prostatectomy
SD: Standard deviation
US: United States

## References

[1] Frankish, K. & Ray, W. M. *The Cambridge Handbook of Artificial Intelligence*(Cambridge University Press, 2014).

[2] Powers, B. J., Trinh, J. V. & Bosworth, H. B. Can this patient read and understand written health information? *JAMA* 304, (2010). 10.1001/jama.2010.896 20606152

[3] Agarwala, A. et al. Implementation of Prevention Science to Eliminate Health Care Inequities in Achieving Cardiovascular Health: A Scientific Statement From the American Heart Association. *Circulation*10.1161/CIR.0000000000001171 (2023). 37698007 10.1161/CIR.0000000000001171

[4] Kutner, M. et al. Literacy in everyday life: results from the 2003 National assessment of adult literacy. NCES 2007 – 490. *National Cent. Educ. Statistics* (2007).

[5] Raychaudhuri, R., Lin, D. W. & Montgomery, R. B. Prostate cancer: A review. *JAMA* **333**, 1433–1446 (2025). 40063046 10.1001/jama.2025.0228

[6] Nedbal, C. et al. ChatGPT in urology practice: revolutionizing efficiency and patient care with generative artificial intelligence. *Curr. Opin. Urol.* **34**, 98–104. 10.1097/MOU.0000000000001151 (2024). 37962176 10.1097/MOU.0000000000001151

[7] Eppler, M. B. et al. Bridging the gap between urological research and patient understanding: the role of large Language models in automated generation of layperson’s summaries. *Urol. Pract.* **10**, 436–443 (2023). 37410015 10.1097/UPJ.0000000000000428

[8] Gabrielson, A. T., Odisho, A. Y. & Canes, D. Harnessing generative artificial intelligence to improve efficiency among urologists: welcome ChatGPT. *J. Urol.* **209**, 827–829 (2023). 36795957 10.1097/JU.0000000000003383

[9] Davis, R. et al. Evaluating the effectiveness of artificial Intelligence-powered large Language models application in disseminating appropriate and readable health information in urology. *J. Urol.* **210**, 688–694 (2023). 37428117 10.1097/JU.0000000000003615

[10] Beaulieu-Jones, B. R. et al. Evaluating Capabilities of Large Language Models: Performance of GPT4 on Surgical Knowledge Assessments. *Surgery*10.1101/2023.07.16.23292743 (2023). 10.1016/j.surg.2023.12.014 PMC10947829 38246839

[11] Jindal, P. & Macdermid, J. C. Assessing reading levels of health information: uses and limitations of Flesch formula. *Educ. Health: Change Learn. Pract.* **30**, 84–88 (2017). 10.4103/1357-6283.210517 28707643

[12] Friedman, D. B. & Hoffman-Goetz, L. A systematic review of readability and comprehension instruments used for print and web-based cancer information. *Health Educ. Behav.* **33**, 352–373. 10.1177/1090198105277329 (2006). 16699125 10.1177/1090198105277329

[13] Musheyev, D., Pan, A., Loeb, S. & Kabarriti, A. E. How well do artificial intelligence chatbots respond to the top search queries about urological malignancies?? *Eur. Urol.* **85**, 13–16 (2024). 37567827 10.1016/j.eururo.2023.07.004

[14] Coskun, B., Ocakoglu, G., Yetemen, M. & Kaygisiz, O. Can ChatGPT, an Artificial Intelligence Language Model, Provide Accurate and High-quality Patient Information on Prostate Cancer?. *Urology* **180**, 35–58 (2023). 37406864 10.1016/j.urology.2023.05.040

[15] Szczesniewski, J. J. et al. ChatGPT and most frequent urological diseases: analysing the quality of information and potential risks for patients. *World J. Urol.* **41**, 3149–3153 (2023). 37632558 10.1007/s00345-023-04563-0

[16] Coughlin, S. S. A review of social determinants of prostate cancer risk, stage, and survival. *Prostate Int.* **8**, 49–54. 10.1016/j.prnil.2019.08.001 (2020). 32647640 10.1016/j.prnil.2019.08.001 PMC7335972

[17] Hamstra, D. A. et al. The impact of numeracy on verbatim knowledge of the longitudinal risk for prostate cancer recurrence following radiation therapy. *Med. Decis. Making*. **35**, 27–36 (2015). 25277673 10.1177/0272989X14551639 PMC4567273

[18] Rock, C. et al. Income level and treatment selection in prostate cancer: analysis of a North Carolina population-based cohort. *JNCI Cancer Spectr* **7**, (2023). 10.1093/jncics/pkad032 PMC10212533 37104733

[19] Holmes-Rovner, M. et al. Evidence-based patient choice: a prostate cancer decision aid in plain Language. *BMC Med. Inf. Decis. Mak.* **5**, 16 (2005). 10.1186/1472-6947-5-16 PMC1168900 15963238

[20] Weiss, B. D. *Health Literacy: A Manual for Clinicians* (American Medical Association Foundation and American Medical Association, 2003).

[21] Rustomji, Y., Nweke, U. C., Hassan, S., Ahmad, U. & Jolly, M. Not so patient friendly: patient education materials in rheumatology and internal medicine fall short of nationally recommended readability benchmarks in the united States. *Arthritis Care Res. (Hoboken)*. **77**, 676–684 (2025). 39609043 10.1002/acr.25473

[22] Jeblick, K. et al. ChatGPT makes medicine easy to swallow: an exploratory case study on simplified radiology reports. *Eur. Radiol.* **34**, 2817–2825 (2024). 37794249 10.1007/s00330-023-10213-1 PMC11126432

[23] Slowik, C. & Kaiser, F. *GPT-3 vs GPT-4: OpenAI Language Models Comparison* (Accessed: 25 July 2025).https://neoteric.eu/blog/gpt-4-vs-gpt-3-openai-models-comparison/(2023).

[24] American Medical Association. Augmented Intelligence in Health Care: AMA Policy Recommendations (Accessed: 25 July 2025).https://www.ama-assn.org/system/files/ama-ai-principles.pdf (American Medical Association, 2024).

[25] Payne, P. R. O. et al. Toward an artificial intelligence code of conduct for health and healthcare: implications for the biomedical informatics community. *J. Am. Med. Inform. Assoc.* **32**, 408–412 (2025). 39658328 10.1093/jamia/ocae306 PMC11756637

[26] World Health Organization. Ethics and Governance of Artificial Intelligence for Health: Guidance on Large Multi-Modal Models. (Accessed: 25 July 2025).https://iris.who.int/bitstream/handle/10665/375579/9789240084759-eng.pdf (World Health & Organization, 2024).

[27] National Academy of Medicine. *An Artificial Intelligence Code of Conduct for Health and Medicine: Essential Guidance for Aligned Action* (National Academies, 2025). 40674514

[28] Dankwa-Mullan, I. Health equity and ethical considerations in using artificial intelligence in public health and medicine. *Prev. Chronic Dis.* **21**, 240245 (2024). 10.5888/pcd21.240245 PMC11364282 39173183

[29] Federation of State Medical Boards. Navigating the Responsible and Ethical Incorporation of Artificial Intelligence into Clinical Practice. (Accessed: 25 July 2025). https://www.fsmb.org/siteassets/advocacy/policies/incorporation-of-ai-into-practice.pdf (Federation of State Medical Boards, 2024).

[30] Suarez-Ibarrola, R., Hein, S., Reis, G., Gratzke, C. & Miernik, A. Current and future applications of machine and deep learning in urology: a review of the literature on urolithiasis, renal cell carcinoma, and bladder and prostate cancer. *World Journal of Urology* **38** 2329–2347 https://doi.org/10.1007/s00345-019-03000-5 (2020). 10.1007/s00345-019-03000-5 31691082

[31] Paasche-Orlow, M. Caring for patients with limited health literacy: A 76-year-old man with multiple medical problems. in *JAMA* 306 (2011). 10.1001/jama.2011.1203 21828309

[32] Mossanen, M. et al. Readability of urologic pathology reports: the need for patient-centered approaches. *Urologic Oncology: Seminars Original Investigations* **32**, (2014). 10.1016/j.urolonc.2014.04.011 24846343

[33] Cascella, M., Montomoli, J., Bellini, V. & Bignami, E. Evaluating the feasibility of ChatGPT in healthcare: an analysis of multiple clinical and research scenarios. *J Med. Syst* **47**, (2023). 10.1007/s10916-023-01925-4 PMC9985086 36869927

[34] Proctor, E. S. et al. Bridging the gap: evaluating ChatGPT-generated, personalized, patient-centered prostate biopsy reports. *Am. J. Clin. Pathol.* **163**, 766–774 (2025). 39838829 10.1093/ajcp/aqae185

[35] Saffar, A. Patient-centred pathology reporting improves patient experience and Understanding of disease in prostate cancer care. *BJUI Compass*. 10.1002/bco2.322 (2024). 38633832 10.1002/bco2.322 PMC11019249

[36] Centers for Disease Control and Prevention. *Simply Put: A Guide for Creating Easy-to-Understand Materials*. 3rd edn. (Accessed: 25 July 2025). https://stacks.cdc.gov/view/cdc/11938 (Centers for Disease Control and Prevention, 2009).

[37] Solomonides, A. E. et al. Defining amia’s artificial intelligence principles. *J. Am. Med. Inform. Assoc.* **29**, 585–591 (2022). 35190824 10.1093/jamia/ocac006 PMC8922174

