# Supplementary material for: Evaluation of prostate cancer pathology reports generated by ChatGPT to enhance patient comprehension
Source: Sci Rep. 2025 Oct 2;15:34459. doi: 10.1038/s41598-025-17639-1 (PMC12491622; doi:10.1038/s41598-025-17639-1)
Supplement: Supplementary file 1 — Supplementary Material 1 [file 41598_2025_17639_MOESM1_ESM.docx]

***Supplementary Material 1:***

**Prostate Biopsy Prompt:**

*“Your task is to generate a patient-centered pathology report for patients with suspected prostate cancer who are receiving a prostate biopsy. Your report should take complex medical information and make it understandable for someone without a medical background. Please explain the purpose of the biopsy and what the results might mean. Please focus on each biopsy core result. Be sure to use clear and empathetic language, while still being accurate and honest. This explanation should be from the perspective of a Urologist. The pathology report is below:”*

***Sample ChatGPT-generated Biopsy Reports:***

**Gleason Grade Group 1:**

*Pathology Report for Prostate Biopsy*

*Dear Patient,*

*I hope this message finds you well. I am Dr. [Your Name], your urologist, and I wanted to personally discuss the results of your recent prostate biopsy. I understand that medical terminology can be overwhelming, so I will explain the findings in a way that is clear and understandable.*

*The purpose of the prostate biopsy is to investigate the presence of any abnormal changes in your prostate gland. This procedure helps us determine if there are any signs of prostate cancer. Your biopsy involved collecting small samples of tissue from different areas of your prostate for analysis.*

*Now, let's discuss the results of each biopsy core individually:*

1. *PROSTATE, LEFT LATERAL BASE, BIOPSY:*

*The tissue sample taken from this area of your prostate showed benign (non-cancerous) prostatic tissue. This means that there are no signs of cancer in this particular area.*

*B. PROSTATE, LEFT LATERAL MID, BIOPSY:*

*Similarly, the tissue sample from the left lateral mid area also revealed benign prostatic tissue, indicating the absence of cancer.*

*C. PROSTATE, LEFT LATERAL APEX, BIOPSY:*

*In this area, a small focus of prostatic adenocarcinoma, which is a type of prostate cancer, was detected. However, it is important to note that this finding is of grade group 1, which means it is a low-grade cancer. The Gleason score, a grading system used to assess prostate cancer, is 3+3=6 in this case. The tumor involves only 1% of the overall tissue, indicating that it is a very small area of cancer. Furthermore, no perineural invasion was identified, which is a positive finding.*

*D. PROSTATE, LEFT MEDIAL BASE, BIOPSY:*

*The biopsy from this region displayed benign prostatic tissue, signifying the absence of cancer.*

*E. PROSTATE, LEFT MEDIAL MID, BIOPSY:*

*Similar to the previous sample, the tissue from the left medial mid area is benign, indicating no cancerous changes.*

*F. PROSTATE, LEFT MEDIAL APEX, BIOPSY:*

*The biopsy core taken from the left medial apex region also showed benign prostatic tissue, indicating the absence of cancer.*

*G. PROSTATE, RIGHT MEDIAL BASE, BIOPSY:*

*The tissue sample from this area of your prostate revealed benign prostatic tissue, indicating no signs of cancer.*

*H. PROSTATE, RIGHT MEDIAL MID, BIOPSY:*

*Similar to the previous findings, the biopsy core taken from the right medial mid region showed a small focus of prostatic adenocarcinoma, grade group 1. The Gleason score is 3+3=6, suggesting low-grade cancer. The tumor involves only 1% of the overall tissue, and no perineural invasion was identified.*

*I. PROSTATE, RIGHT MEDIAL APEX, BIOPSY:*

*The biopsy core taken from the right medial apex region revealed benign prostatic tissue, indicating the absence of cancer.*

*J. PROSTATE, RIGHT LATERAL BASE, BIOPSY:*

*The tissue sample collected from this area showed benign prostatic tissue, indicating no signs of cancer.*

*K. PROSTATE, RIGHT LATERAL MID, BIOPSY:*

*Similarly, the biopsy core from the right lateral mid region displayed benign prostatic tissue, suggesting the absence of cancer.*

*L. PROSTATE, RIGHT LATERAL APEX, BIOPSY:*

*In this region, the biopsy core revealed prostatic adenocarcinoma, grade group 1. The Gleason score is 3+3=6, indicating low-grade cancer. The tumor involves 10% of the overall tissue, which is still a relatively small area. Additionally, no perineural invasion was identified.*

*M. PROSTATE, PIRADS 3 (X3), BIOPSY:*

*The biopsy sample from this area showed benign prostatic tissue, indicating no signs of cancer.*

*In conclusion, the results of your prostate biopsy indicate a mixed pattern of findings. While the majority of the biopsy cores showed benign prostatic tissue without any evidence of cancer, there were small foci of low-grade prostatic adenocarcinoma in two areas: the left lateral apex and the right lateral apex. It's important to note that these cancerous areas represent a very small percentage of the overall tissue. Additionally, the absence of perineural invasion is an encouraging finding.*

*I understand that this information may be overwhelming, but it is crucial to remember that the detected cancers are low-grade and localized. We will discuss the treatment options available to you and develop a comprehensive plan to ensure your well-being.*

*Please do not hesitate to reach out with any questions or concerns you may have. We are here to support you throughout your healthcare journey.*

*Take care and warm regards,*

*[Your Name]*

**Gleason Grade Group 3:**

*Pathology Report Explanation for Prostate Biopsy*

*Dear Patient,*

*I have carefully reviewed the results of your recent prostate biopsy, and I want to provide you with a clear understanding of the findings. The purpose of the biopsy was to investigate your prostate gland for any signs of cancer, as indicated by your elevated PSA levels.*

*Let's go through each biopsy core result individually:*

*A. PROSTATE, LEFT LATERAL BASE #1, BIOPSY:*

*The tissue sample taken from the left lateral base of your prostate gland shows benign prostatic tissue. This means that no cancerous cells were found in this specific area.*

*B. PROSTATE, LEFT LATERAL MID #2, BIOPSY:*

*Similarly, the biopsy from the left lateral mid area reveals benign prostatic tissue, indicating the absence of cancerous cells in this region as well.*

*C. PROSTATE, LEFT LATERAL APEX #3, BIOPSY:*

*The sample obtained from the left lateral apex of your prostate gland demonstrates benign prostatic tissue, indicating no presence of cancerous cells in this area.*

*D. PROSTATE, LEFT MEDIAL BASE #4, BIOPSY:*

*The biopsy core from the left medial base of your prostate gland exhibits benign prostatic tissue, indicating the absence of cancerous cells in this particular location.*

*E. PROSTATE, LEFT MEDIAL MID #5, BIOPSY:*

*Similar to the previous results, the biopsy from the left medial mid area shows benign prostatic tissue, indicating the absence of cancer cells in this region as well.*

*F. PROSTATE, LEFT MEDIAL APEX #6, BIOPSY:*

*The tissue sample taken from the left medial apex region demonstrates benign prostatic tissue, indicating no presence of cancer cells in this area.*

*G. PROSTATE, RIGHT MEDIAL BASE #7, BIOPSY:*

*The biopsy core obtained from the right medial base of your prostate gland reveals benign prostatic tissue, indicating the absence of cancerous cells in this specific location.*

*H. PROSTATE, RIGHT MEDIAL MID #8, BIOPSY:*

*Similar to the previous results, the biopsy from the right medial mid area shows benign prostatic tissue, indicating the absence of cancerous cells in this region as well.*

*I. PROSTATE, RIGHT MEDIAL APEX #9, BIOPSY:*

*The sample taken from the right medial apex of your prostate gland exhibits benign prostatic tissue, indicating no presence of cancer cells in this area.*

*J. PROSTATE, RIGHT LATERAL BASE #10, BIOPSY:*

*In the biopsy core obtained from the right lateral base, benign fibromuscular tissue is identified. This is a non-cancerous tissue type and not indicative of prostate cancer.*

*K. PROSTATE, RIGHT LATERAL MID #11, BIOPSY:*

*The biopsy from the right lateral mid area shows the presence of prostatic adenocarcinoma, which is a type of prostate cancer. The grade group is 3, which indicates the aggressiveness of the cancer. The Gleason score, a grading system for prostate cancer, is 4+3=7, with pattern 4 accounting for 70% of the cancerous cells. Perineural invasion, where cancer cells invade the nerves, is identified, which may affect treatment decisions. The tumor involves 30% of the overall tissue in this area.*

*L. PROSTATE, RIGHT LATERAL APEX #12, BIOPSY:*

*In the biopsy core obtained from the right lateral apex, prostatic adenocarcinoma is also found. However, in this case, the grade group is 2, indicating a less aggressive form of cancer. The Gleason score is 3+4=7, with pattern 4 accounting for 5% of the cancerous cells. No perineural invasion is identified. The tumor involves 30% of the overall tissue in this area.*

*M. PROSTATE, LEFT POSTERIOR PERIPHERAL ZONE MID (X3), BIOPSY:*

*The three biopsy cores taken from the left posterior peripheral zone mid demonstrate benign prostatic tissue, indicating no presence of cancerous cells in this particular region.*

*N. PROSTATE, RIGHT POSTERIOR PERIPHERAL ZONE APEX (X3), BIOPSY:*

*Among the three biopsy cores obtained from the right posterior peripheral zone apex, one of them shows prostatic adenocarcinoma. The grade group is 2, with a Gleason score of 3+4=7, and pattern 4 accounting for 10% of the cancerous cells. No perineural invasion is identified. The tumor involves 40% of the overall tissue in this area.*

*To summarize, the majority of the biopsy cores indicate the presence of benign prostatic tissue, which means no cancerous cells were found in those areas. However, there are specific areas, such as the right lateral mid (core #11) and right lateral apex (core #12), where prostatic adenocarcinoma, a type of prostate cancer, was detected. The grade and Gleason score provide information about the aggressiveness of the cancer, and the involvement of the overall tissue helps determine the extent of the cancer in those areas.*

*Please keep in mind that these biopsy results provide valuable information for us to understand the nature of your prostate condition. However, a comprehensive discussion about your diagnosis, treatment options, and further steps is crucial. I recommend scheduling an appointment with me to discuss these findings in detail and develop an appropriate management plan personalized to your situation.*

*I understand that receiving such news can be overwhelming, but please remember that you are not alone. We are here to support you through this journey and provide you with the best care possible.*

*If you have any immediate concerns or questions, please don't hesitate to contact our clinic. We are committed to your well-being and will assist you in any way we can.*

*Warm regards,*

*[Your Name]*

**Supplementary Material 2:**

**Surgical Pathology Prompt:**

“Your task is to generate a patient-centered pathology report for patients with prostate cancer who have received radical prostatectomy. Your report should take complex medical information and make it understandable for someone without a medical background. Please explain the purpose of the surgical pathology report and what the results might mean. Please focus on the results regarding the Gleason scores, pathological stage, extraprostatic extension, surgical margins, seminal vesicle invasion, and lymph node involvement. Be sure to use clear and empathetic language while still being accurate and honest. This explanation should be from the perspective of a Urologist. The pathology report is below:”

**Sample ChatGPT-generated Surgical Pathology Reports:**

**Report with negative surgical margins:**

*Pathology Report Explanation:*

*Dear Patient,*

*I hope this message finds you well. I am writing to provide you with the results of your recent radical prostatectomy and to explain what they mean for you. As your urologist, it is important for me to ensure that you understand the information in your pathology report.*

*The purpose of the surgical pathology report is to provide a detailed analysis of the tissues and lymph nodes that were removed during your surgery. It helps us determine the extent and characteristics of your prostate cancer and guide your further treatment options. Now, let's go through the different sections of the report.*

*A. Anterior Prostatic Fat:*

*The tissue sample taken from the anterior prostatic fat shows benign fibroadipose tissue. This means that there are no cancer cells present in this area.*

*B. Left Pelvic Lymph Node:*

*Three lymph nodes from the left pelvic area were examined, and all of them are negative for the presence of adenocarcinoma, which is the type of cancer you have. This means that the cancer has not spread to these lymph nodes.*

*C. Right Pelvic Lymph Node:*

*Four lymph nodes from the right pelvic area were examined, and all of them are also negative for adenocarcinoma. This is another positive finding, indicating that the cancer has not spread to these lymph nodes either.*

*D. Prostate, Seminal Vesicles, and Vas Deferens (Radical Prostatectomy):*

*This is the most crucial part of the report as it describes the findings from your prostate and surrounding structures.*

*1. Prostatic Adenocarcinoma, Gleason Score 3+4=7 (Grade Group 2):*

*The pathology analysis confirms the presence of prostate cancer in your prostate gland. It is a mixed type, comprising both acinar and ductal components. The Gleason score, which helps us determine the aggressiveness of the cancer, is 3+4=7. This means that the cancer cells have characteristics corresponding to Gleason pattern 3 and Gleason pattern 4. The higher the Gleason score, the more aggressive the cancer. However, Grade Group 2 suggests that your cancer is moderately aggressive.*

*2. Organ Confined (pT2):*

*The cancer is classified as "organ confined," which means it is limited to the prostate gland and has not spread beyond its boundaries. This is an encouraging finding.*

*3. Surgical Margins: Uninvolved by Carcinoma:*

*The surgical margins, which refer to the outer edges of the removed prostate tissue, are uninvolved by carcinoma. In other words, there is no evidence of cancer cells at the edges. This is a positive outcome, as it indicates that the surgeon was able to remove the entire cancerous tissue during the operation.*

*4. CAP Cancer Protocol Summary - Prostate:*

*This section provides additional details about the characteristics of your cancer. It mentions that the cancer is predominantly composed of the acinar type, with a small percentage (20%) showing a cribriform pattern. It also notes the absence of intraductal carcinoma, which is a type of cancer that can sometimes coexist with prostate adenocarcinoma.*

*5. Additional Pathologic Findings:*

*Apart from the cancer, the report mentions the presence of high-grade prostatic intraepithelial neoplasia (PIN). PIN is a precancerous condition that may indicate an increased risk of developing prostate cancer in the future. It also notes the presence of benign prostatic hyperplasia (enlarged prostate) and acute and chronic inflammation. These findings are not uncommon and often coexist with prostate cancer.*

*6. Lymph Node Involvement:*

*The lymph nodes that were examined during your surgery, both in the left and right pelvic areas, show no*

*evidence of cancer involvement. This means that the cancer has not spread to the lymph nodes, which is a positive finding.*

*In summary, based on the pathology report, your prostate cancer has been characterized as moderately aggressive (Grade Group 2) with a Gleason score of 3+4=7. The cancer is organ confined (pT2), and the surgical margins are clear, indicating successful removal of the cancerous tissue. The lymph nodes examined are negative for cancer involvement. This means that the cancer has not spread beyond the prostate, increasing the chances of a successful treatment outcome.*

*I understand that receiving this diagnosis and going through surgery can be overwhelming. It is important to remember that this report provides crucial information to guide your treatment plan and ensure the best possible outcome. We will discuss these results in detail during your next appointment, and together, we will develop a comprehensive plan tailored to your specific needs.*

*Please do not hesitate to reach out to me if you have any questions or concerns. I am here to support you throughout your treatment journey.*

*Take care and stay positive.*

*Warm regards,*

*[Your Name]*

*Urologist*

**Report with positive surgical margins:**

*Pathology Report: Radical Prostatectomy Results*

*Pathology Report Summary - Prostate Cancer Patient*

*Dear [Patient Name],*

*I hope this message finds you well. I am writing to provide you with the results of your recent pathology report following your radical prostatectomy. As your urologist, it is my responsibility to explain these findings to you in a way that is easy to understand. The pathology report helps us understand the nature of your prostate cancer and its characteristics to guide further treatment decisions.*

*First, let's go over the purpose of the surgical pathology report. After your prostatectomy, the removed tissues were examined under a microscope by a pathologist who specializes in diagnosing diseases. The report provides crucial information about the cancer, including its grade, stage, and involvement of surrounding tissues. This information helps us determine the best course of action for your treatment and prognosis.*

*Now, let's discuss the key findings from your report:*

*1. Gleason Score and Grade Group: The Gleason score is a measure of how aggressive the cancer cells appear under the microscope. Your Gleason score is 4+5=9, which indicates a high-grade cancer. The Grade Group associated with this score is 5. This means that the cancer cells have a high likelihood of growing and spreading.*

*2. Pathological Stage: The pathological stage classifies the extent of cancer based on how far it has spread beyond the prostate gland. In your case, the cancer has invaded the seminal vesicles, which are structures adjacent to the prostate. Therefore, your stage is classified as pT3b.*

*3. Extraprostatic Extension: The report indicates that the cancer has spread beyond the prostate gland into the surrounding tissues. Specifically, it involves both seminal vesicles as well as the left and right posterior and anterior regions of the prostate.*

*4. Surgical Margins: The surgical margins refer to the edges of the tissue that were removed during the surgery. In your case, there are positive margins, indicating the presence of cancer cells close to the edges. However, the extent of involvement is small, measuring less than 1 millimeter to focal 2 millimeters. The positive margins are located in the right anterior and left anterior regions of the bladder neck.*

*5. Seminal Vesicle Invasion: The cancer has invaded the seminal vesicles, which are structures responsible for producing semen.*

*6. Lymph Node Involvement: The report states that one lymph node was examined, and it is negative for cancer cells. This suggests that the cancer has not spread to the lymph nodes.*

*Understanding these findings is essential for determining the most appropriate treatment plan. It is important to note that your cancer is aggressive and has involved the seminal vesicles and surrounding tissues. We will carefully consider these factors when discussing further treatment options.*

*I understand that this information may be overwhelming, but please know that we are here to support you throughout this journey. It is crucial to schedule a follow-up appointment with me to discuss these results in detail, address any questions or concerns you may have, and develop an individualized treatment plan.*

*Take care, and I look forward to meeting with you soon.*

*Sincerely,*

*[Your Name]*

*Urologist*
